# Fatuity

**Published:** 1870-12

**Authors:** 


### FATUITY

Is weak-mindedness, imbecility ; that form of it which is the result of old age, manifests itself in a want of memory, which causes many foolish and silly things to be said and done ; there is also a want of judgment in many of the common affairs of life, and the mind seems to have lost its balance as to the most ordinary transactions, while on a few subjects—that one subject in each, which has been the ruling passion of life—there is a degree of acuteness, the more marvellous from its contrast, with an almost entire absence of sense in other things. Some call it childishness, which, minds approaching it, regard with peculiar horror ; just as persons whose dread of cancer is almost the terror of life, do at length become its victims.

For the fatuity of old age there is no cure, because the state of mind which induced it has worked organic changes in the brain ; has in a sense changed the nature of its substance, as life-long scars are left as effects of wounds on the body. Fatuity may be modified, may be kept at bay to a certain extent, so as to make comparatively a very slow progress, by a simple effort of the will in cultivating company, and lively conversation. Encourage visiting ; go and see your friends every day if possible, and make friends happy who come to see you—

then they will come oftener—the happiness which arises from a sincerity of reception, where there is a true, whole-heartedness, no sham, no pretence, no hypocrisy.

Fatuity may be prevented by following the opposite of the things which bring it on. The chief causes of fatuity are—

1st. Living a secluded life ; some persons have no sociability ; they do not go to see others ; they do not want to have others come and see them. This is generally the result of intense selfishness ; they are so immersed in their own affairs, so busy in their own interests, that they do not like to be interrupted ; hence, there is more or less of coldness, or reserve, and that visitor comes no more ; or if there is an occasional call, it is stiff and formal ; there is no going out of heart-to-heart ; there is no reciprocity of frankness and true friendship ; and soon the heart becomes an icicle, and the affections are dead. Thus it is that there are not a few who die, and not a single human being sheds a tear at the grave ; not only no regret is felt at the final passing away, but there is an absolute feeling of relief at the riddance ; the very contemplation of such an ending is positively terrible.

2d. There are others of the old, who arrive at the same destination from a miserly disposition, practiced in youth from necessity it may be, and in later life from the sheer greed of gold. The habit of saving becomes so infixed, that it is impossible to break it up ; it is a second nature, an uncontrollable passion ; it becomes meat and drink, and existence itself ; everything is swallowed up in it, is sacrificed to it, and all sympathy, friendship, affection, love, and even a common humanity, are irrevocably dead.

As we grow older, then, let us practice benevolence more ; cultivate geniality, social intercourse, and a generous reciprocity of all the sweet courtesies of social and domestic life, and then tears and flowers will be mingled at our grave, and the memory of us will be blessed.

